# Placental Small Extracellular Vesicles Undetected in Cerebrospinal Fluid of Preeclamptic and Eclamptic Women

**DOI:** 10.3390/biom16081211

**Published:** 2026-08-19

**Authors:** Bryony Davies, Faheem Seedat, Lina Bergman, Catherine Cluver, Angga Wiratama Lokeswara, Michelle Ma, Morganne Wilbourne, Shuhan Jiang, Antonio Galvez, Adam Handel, Andrew Fower, Carlos Escudero, Wei Zhang, Manu Vatish

**Affiliations:** 1 Women’s Centre, Nuffield Department of Women’s & Reproductive Health, John Radcliffe Hospital, University of Oxford, Oxford OX3 9DU, UK; bryony.davies@wrh.ox.ac.uk (B.D.); faheem.seedat@wrh.ox.ac.uk (F.S.); angga.lokeswara@wrh.ox.ac.uk (A.W.L.); michelle.ma@wrh.ox.ac.uk (M.M.); morganne.wilbourne@spc.ox.ac.uk (M.W.); shuhan.jiang@mpl.mpg.de (S.J.); cescudero@ubiobio.cl (C.E.); wei.zhang@wrh.ox.ac.uk (W.Z.); 2 SAMRC Preeclampsia Extramural Research Unit, Department of Obstetrics and Gynaecology, Stellenbosch University, Cape Town 7500, South Africa; lina.bergman@obgyn.gu.se (L.B.); cathycluver@sun.ac.za (C.C.); 3 Department of Women’s and Children’s Health, Uppsala University, SE-751 85 Uppsala, Sweden; 4 Department of Obstetrics and Gynaecology, Institute of Clinical Sciences, Sahlgrenska Academy, University of Gothenburg, 41685 Gothenburg, Sweden; 5 Translational Obstetrics Group, Department of Obstetrics and Gynaecology, University of Melbourne, Melbourne, VIC 3010, Australia; 6 Mercy Perinatal, Mercy Hospital for Women, Heidelberg, VIC 3084, Australia; 7 Max Planck Institute for The Science of Light, 91058 Erlangen, Germany; 8 Anesthesiology Department, Hospital Clinico Herminda Martin de Chillan, Chillan 3810525, Chile; antonio.galvez.verdugo@gmail.com; 9 Autoimmune Neurology Group, Nuffield Department of Clinical Neurosciences, John Radcliffe Hospital, University of Oxford, West Wing, Oxford OX3 9DU, UK; adam.handel@ndcn.ox.ac.uk (A.H.); andrew.fower@ndcn.ox.ac.uk (A.F.); 10 Group of Research and Innovation in Vascular Health, Department of Basic Science, Faculty of Sciences, Universidad del Bio Bio, Chillan 3810189, Chile

**Keywords:** extracellular vesicles, cerebrospinal fluid, blood–brain barrier, preeclampsia, eclampsia, hypertensive disorders of pregnancy

## Abstract

Circulating small extracellular vesicles (sEVs) released from the placenta carry bioactive compounds. Placental sEVs (psEVs) have been implicated as drivers of pathology in preeclampsia, a common disorder of pregnancy. This study investigates the hypothesis that psEVs are detectable in cerebrospinal fluid (CSF) during pregnancy, and are present at higher concentrations in preeclamptic and eclamptic pregnancies. Two techniques were used to search for psEVs. Firstly, the ExoCounter assay was performed on neat CSF from normotensive, preeclamptic and eclamptic pregnancies, and non-pregnant controls (*n* = 11, 12, 10 and 4, respectively). Quantitative PCR was used to search for psEV-associated microRNAs in the CSF of pregnant women. Neither assay found evidence of psEVs in the CSF of pregnant women, regardless of whether they had preeclampsia or eclampsia. This study suggests that psEVs do not reside in CSF during pregnancy and may be more likely to impact central nervous tissues through peripheral changes or interaction with the blood–brain barrier without crossing.

## 1. Introduction

Preeclampsia is a common syndrome of pregnancy, defined by new-onset hypertension accompanied by new-onset end-organ complications [1]. Preeclampsia can cause neurological complications, including seizure (eclampsia), cortical blindness, ischemic stroke, and subarachnoid or intracerebral haemorrhage [2,3]. These complications represent a significant clinical burden and are estimated to cause 30–70% of preeclampsia-linked maternal deaths [3,4,5]. Preeclampsia is associated with increased risk of future neurological disorders, suggesting potential long-term changes [3].

The mechanisms underlying preeclamptic neuropathology, particularly seizure, remain unclear. Early theories linked seizure with poor adaptation to hypertension but are challenged by the poor correlation between hypertension and seizure risk and cases of seizure occurrence in the absence of hypertension [6,7,8]. There is evidence of blood–brain barrier (BBB) injury and neuroinflammation in eclampsia, but the factors contributing to this state remain unclear [9,10,11]. Limited understanding of underlying mechanisms has hindered the development of therapies against preeclamptic neuropathology.

Small extracellular vesicles (sEVs) are lipid-bound particles released from cells into extracellular space, enabling communication between physically distant tissues. sEVs contain biologically active cargo, including proteins, nucleic acids and metabolites, that can be released into recipient cells by internalisation or fusion with the plasma membrane [12,13]. sEVs are differentiated from other extracellular vesicle (EV) subcategories by having a diameter below 200 nm. During pregnancy, placental sEVs (psEVs) are released from the syncytiotrophoblast layer of the placenta into maternal circulation [14].

psEVs have been implicated as drivers of preeclamptic pathology. Preeclamptic psEVs are enriched in molecules with anti-angiogenic properties, such as neprilysin and soluble fms-like tyrosine kinase-1, and show reduced levels of endothelial nitric oxide synthase [15,16]. Vascular endothelium exposed to preeclamptic psEVs shows structural damage, implicating psEVs in endothelial pathology observed in preeclampsia [17,18]. Furthermore, preeclamptic psEVs exert pro-inflammatory effects on macrophages and endothelial cells and may modulate lymphocyte and monocyte responses, thus contributing to systemic inflammation in preeclampsia [19,20,21,22].

The central nervous system is protected by specialised neurovascular interfaces, including the BBB and the blood–CSF barrier at the choroid plexus. CSF is predominantly produced by the choroid plexus, a highly vascularized structure located within the cerebral ventricles. Choroid plexus capillaries are fenestrated and relatively permeable, facilitating exchange between circulation and CSF [23]. Emerging evidence suggests that circulating sEVs of non-preeclamptic origin can cross the BBB and blood–CSF barrier, particularly in inflammatory contexts [24,25,26].

Several lines of evidence support the hypothesis that psEVs may cross the BBB and/or the blood–CSF barrier: (i) the permeability of choroid plexus capillaries, (ii) evidence of non-preeclamptic sEVs crossing the BBB and blood–CSF barrier, and (iii) a recent study demonstrating that circulating sEVs obtained from preeclamptic women were able to cross an in vitro brain endothelial cell barrier [23,24,25,26,27].

In vitro BBB models show increased permeability after exposure to plasma or psEVs from women with preeclampsia or eclampsia, compared to normotensive women [28,29]. Circulating numbers of psEVs are higher in preeclamptic women, and BBB and blood–CSF barrier permeability is increased in states of systemic inflammation, such as preeclampsia [2,23,26]. Thus, it is plausible that psEV crossing of the BBB or blood–CSF barrier would be increased in preeclampsia and eclampsia. However, this possibility has not been investigated in humans.

The ExoCounter assay combines size-based exclusion with dual-antibody detection, enabling quantification of specific sEV subpopulations [13,30]. Using CD63 as a general sEV marker and placental alkaline phosphatase (PLAP) as a marker of placental origin, the ExoCounter assay can quantify psEVs in plasma [13].

This study addresses the hypothesis that psEVs are present in CSF, with numbers increased during preeclampsia and eclampsia. The study’s aim was quantification and comparison of psEV numbers in the CSF of women with normotensive pregnancy, preeclampsia or eclampsia, and non-pregnant controls. To achieve this, the ExoCounter assay was validated for use in CSF and applied to CSF samples from normotensive, preeclamptic, eclamptic and non-pregnant women. This was supplemented by quantitative PCR (qPCR) of psEV-associated miRNA clusters in a subset of CSF samples. To our knowledge, this is the first study to investigate psEVs in human CSF.

## 2. Materials and Methods

### 2.1. Setup of Methodology for psEV Quantification in CSF

The ExoCounter assay (JVCKENWOOD Corp., Yokohama, Japan) was used to quantify psEVs in CSF because of its low detection limit and ability to analyse small sample volumes [13,30]. The assay was performed as described by Jiang et al. [13], using 20 μg/mL of anti-CD63 antibody (BioLegend Ultra-LEAF Purified anti-human CD63 Antibody, 353040) and 20 μg/mL of anti-PLAP antibody (NDOG2, in-house) conjugated with magnetic beads (JVCKENWOOD). An amount of 50 μL of sample was added per well; CSF samples were run neat. Samples were analysed in triplicate; a fourth well was used for background measurement without anti-CD63 antibody. Corrected sEV counts were calculated by subtracting the background count from the mean count of the triplicate. Where the background count is greater than the observed count, this is recorded as 0.

Plasma samples were diluted to a 1-to-4 ratio with PBST (phosphate-buffered saline with 0.05% Tween20 (Sigma Aldrich, Burlington, VT, USA)) before measurement. For the assessment of background binding, CSF was EV-depleted by centrifugation through an Amicon Ultra Centrifugal Filter (Merck, Darmstadt, Germany), 10 kDa MWCO, spun at 14,000× *g* for 20 min at 4 °C.

Suitability of the anti-PLAP antibody (NDOG2) for use in neurological tissue was assessed by quantified Western blotting against non-pregnant plasma (Medical Sciences Interdivisional Research Ethics Committee Ref 2033823), non-pregnant CSF (BioIVT, Human Cerebrospinal Fluid (Remnant) No Diagnosis HUMANCSFR-0101364) and non-pregnant brain lysate (BioTechne, Human Brain Whole Tissue Lysate (Adult Whole Normal NB820-59177)), with placental lysate and psEVs used as positive controls. Western blotting was performed as previously described [14].

psEVs used for methodology setup were obtained by dual-lobe placental perfusion and ultracentrifugation [14]. Isolated psEVs were characterised by nanoparticle tracking analysis (NTA), transmission electron microscopy (TEM) and Western blotting for known placental markers, as previously described [14]. NTA was performed using the NanoSight NS500 system equipped with a 405 nm laser and sCMOS (Malvern Instruments, Malvern, UK). For TEM, particles were diluted with filtered phosphate-buffered saline (PBS) to a protein concentration of 0.1–0.3 µg/µL. Ten microlitres was added onto freshly glowing discharged carbon formvar 300 mesh copper grids for 2 min, dried using filter paper, stained with 2% uranyl acetate for 10 s, and left to air-dry. The grid was negatively stained to enhance contrast. Imaging was completed using an FEI Tecnai TEM at 120 kV with a Gatan OneView CMOS camera (Sir William Dunn School of Pathology, University of Oxford, Oxford, UK). Western blots were imaged with the iBright 1000 Imaging System (ThermoFisher Scientific, Waltham, MA, USA). psEVs were blotted with antibodies against CD63 (AbCam EPR5702), syntenin (AbCam AB133267), Alix (Novus Biologicals NBP1-49701), and cytochrome c (Santa Cruz sc-13156).

Linearity of the ExoCounter assay was tested by serial dilution of exogenous psEVs into non-pregnant CSF. Dependence of the assay on intact vesicular structure was assessed by incubating psEVs with 1% Nonidet P-40 (NP-40) for 1 h (ThermoFisher Scientific, USA) in filtered PBST.

### 2.2. Assessment of Normotensive, Preeclamptic and Eclamptic CSF Samples

Women with normotensive pregnancy (*n* = 11), preeclampsia (*n* = 12), and eclampsia (*n* = 10) undergoing a caesarean section with spinal anaesthesia were recruited to the Preeclampsia Obstetric Adverse Events Biobank at Tygerberg Hospital, Cape Town, South Africa [31] (REF N17/05/048). Following the PROVE protocol [31], women unable to give informed consent were excluded. Women with pre-existing hypertension; diabetes mellitus before or during their pregnancy; and pre-existing cardiovascular, cerebral or renal disease were excluded. All participants underwent either emergency or elective caesarean section.

Preeclampsia was defined according to the American College of Obstetricians and Gynaecologists’ criteria, but significant proteinuria was also required (protein-to-creatinine ratio of ≥30 mg/mmol [0.3 mg/mg] or ≥0.3 g protein in a 24 h urine collection or a urine dipstick of >1 on multiple occasions). Eclampsia was diagnosed when generalised tonic–clonic seizures occurred in a woman previously diagnosed with preeclampsia in the absence of other aetiologies. Severe hypertension was defined as systolic blood pressure ≥ 160 mmHg and/or diastolic blood pressure ≥ 110 mmHg. Women with eclampsia or preeclampsia were sub-divided into ‘early-onset disease’ (delivery before 34 gestational weeks) or ‘late-onset disease’ (delivery after 34 gestational weeks). Delivery was used as a proxy measure for disease onset as timing of onset cannot be accurately determined, although it is noted that use of gestational age at delivery for categorisation also carries inaccuracies [2,32].

### 2.3. CSF Collection and Analysis

CSF samples were obtained by lumbar puncture at the time of caesarean section and transferred into Sarstedt polypropylene tubes. If visibly contaminated by blood, CSF was discarded. Samples were centrifuged at 2200× *g* for 10 min at 20 °C, aliquoted into cryotubes (Sarstedt, Numbrecht, Germany) and frozen at −80 °C. Research staff were always present when CSF samples were obtained, so all CSF samples were frozen within 2 h of being drawn.

CSF samples were analysed by ExoCounter assay, which was supplemented by quantification of the chromosome 19 microRNA cluster, which is almost exclusively expressed in the placenta [33]. RNA was isolated from 1 mL of CSF and 500 µg of isolated psEVs, diluted to 1 mL. The exoRNAeasy Midi Kit (Qiagen, Venlo, Netherlands) was used for RNA extraction according to the manufacturer’s protocol. This kit selects for EV-associated RNA, so vesicle-free RNA was not analysed.

### 2.4. qPCR for Analysis of Placenta-Specific microRNAs

Following RNA extraction, the quality of eluted RNA was assessed by measuring the 260/280 and 260/230 ratios with a NanoDrop™ 8000 spectrophotometer (ThermoFisher Scientific, USA). RNA concentrations were determined using the Qubit^®^ 2.0 fluorometer (ThermoFisher Scientific, USA) along with the Qubit™ RNA HS Assay Kit (Thermo Fischer Scientific, USA), while microRNA was quantified separately with the Qubit™ microRNA Assay Kit (Thermo Fischer Scientific, USA).

cDNA was synthesised from 10 ng of RNA using the TaqMan Advanced miRNA cDNA Synthesis Kit (ThermoFisher Scientific, USA). Reagents were added according to the manufacturer’s protocol, and reactions were carried out in a thermal cycler under the specified conditions. cDNA concentration was quantified using the Qubit^®^ 2.0 fluorometer and Qubit™ dsDNA HS Assay Kit.

Diluted and concentrated cDNA was analysed in separate assays. cDNA was diluted at 1:10, 1:100, and 1:1000 ratios. Undiluted cDNA at different concentrations (1 µL and 4.5 µL) were also analysed. cDNA was combined with TaqMan™ Fast Advanced Master Mix (2×) (ThermoFisher Scientific, USA), TaqMan™ primers (20×), and nuclease-free water (Invitrogen, Paisley, UK) to reach a total reaction volume of 10 µL per well on the reaction plate. When 4.5 µL of cDNA was used, no nuclease-free water was added. The following TaqMan miRNA assays were analysed: Homo sapiens miR-517a (hsa-miR-517a) (Applied Biosystems, ID: 002402), hsa-miR-517c (Applied Biosystems, ID: 001153), hsa-miR-518b (Applied Biosystems, ID: 001156), and hsa-miR-519a (Applied Biosystems, ID: 001973). All reactions were conducted in triplicate. The analysis was carried out on the QuantStudio™ 6 Flex Real-Time PCR system (ThermoFisher Scientific, USA), and the reverse transcription quantitative PCR (RT-qPCR) was carried out according to the manufacturer’s guidelines. Cq values were obtained using the QuantStudio™ 6 Flex Real-Time PCR system software, Design & Analysis (v2.6.0).

### 2.5. Statistical Analysis

Statistical testing was performed using SPSS Statistics for Windows, Version 29.0.0.0 (SPSS Inc., Chicago, IL, USA), and GraphPad Prism Version 10.2.0 (GraphPad Software Inc., San Diego, CA, USA) software. Where two continuous variables were being compared, Mann–Whitney U test was used. To compare more than two continuous variables, either ANOVA or the Kruskal–Wallis test was used, depending on normality. To assess categorical variables, Chi-square test of independence was used where sample sizes were sufficient; otherwise, Fisher’s exact test was used.

## 3. Results

### 3.1. Setup of Methodology for Quantifying psEVs in CSF

**Figure 1:**
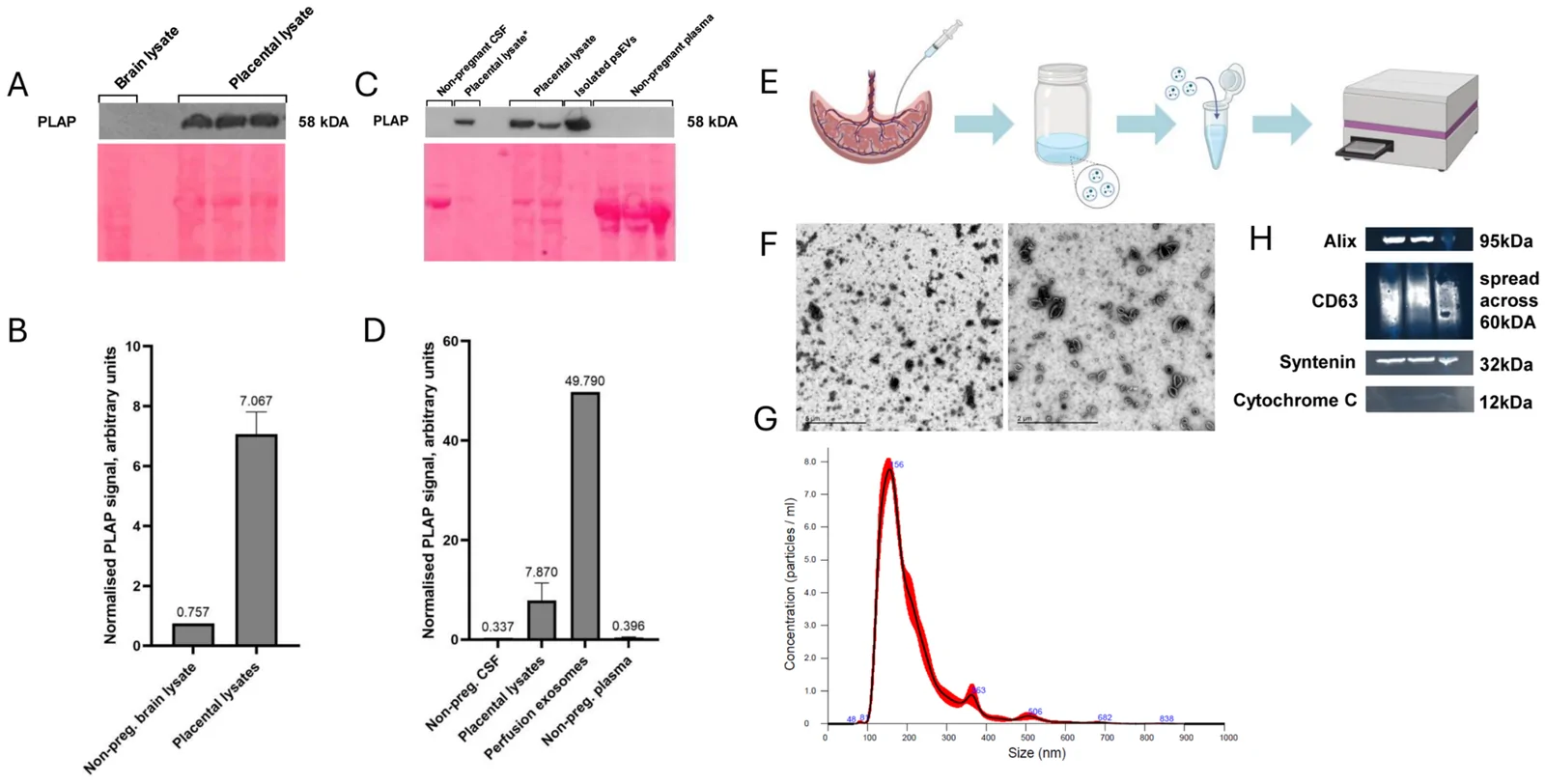
Characterising isolated psEVs. (**A**) Representative images of PLAP identification by Western blotting non-pregnant brain lysate and placental lysate. Ponceau S stain was used as a loading control. (**B**) The quantified PLAP signals of the samples from (**A**) normalised to total protein (Ponceau S stain). Error bars represent standard deviation. (**C**) Representative images of PLAP as in (**A**) using non-pregnant CSF, non-pregnant plasma, isolated psEVs and placental lysate. The asterisk denotes placental lysate that has been diluted to the same low protein concentration as human CSF. (**D**) The quantified PLAP signals of the samples from (**C**). Error bars represent standard deviation. (**E**) Diagram showing the procedure for isolation of psEVs from fresh placentae by perfusion and ultracentrifugation, and spiking of psEVs into non-pregnant CSF samples for measurement by ExoCounter. (**F**) Representative TEM images of isolated psEVs. (**G**) Graph showing the distribution of particle diameters in the isolated psEV sample, as determined by NTA. (**H**) Western blots for sEV markers, including Alix, CD63 and syntenin. Cytochrome C was used as a negative marker of sEVs. See Supplementary Materials for the original images.

The NDOG2 antibody was tested for reactivity against non-pregnant CSF and tissues that contribute to the formation of CSF (peripheral plasma and brain tissue) by quantified Western blotting. The normalised results of western blotting demonstrated no reactivity of the NDOG2 antibody with non-pregnant brain lysate, CSF or plasma. Positive controls (placental lysate and psEVs) showed a strong signal (Figure 1A–D).

psEVs were isolated by placental perfusion and ultracentrifugation for use in methodology setup and optimisation (Figure 1E). Isolated psEVs showed characteristic sEV morphology when visualised by TEM (Figure 1F). Western blotting showed strong signals for sEV marker proteins (Alix, CD63 and syntenin) and no signal for a negative marker of sEVs (cytochrome C) (Figure 1H). NTA demonstrated that the diameter of isolated particles was consistent with sEVs (Figure 1G).

**Figure 2:**
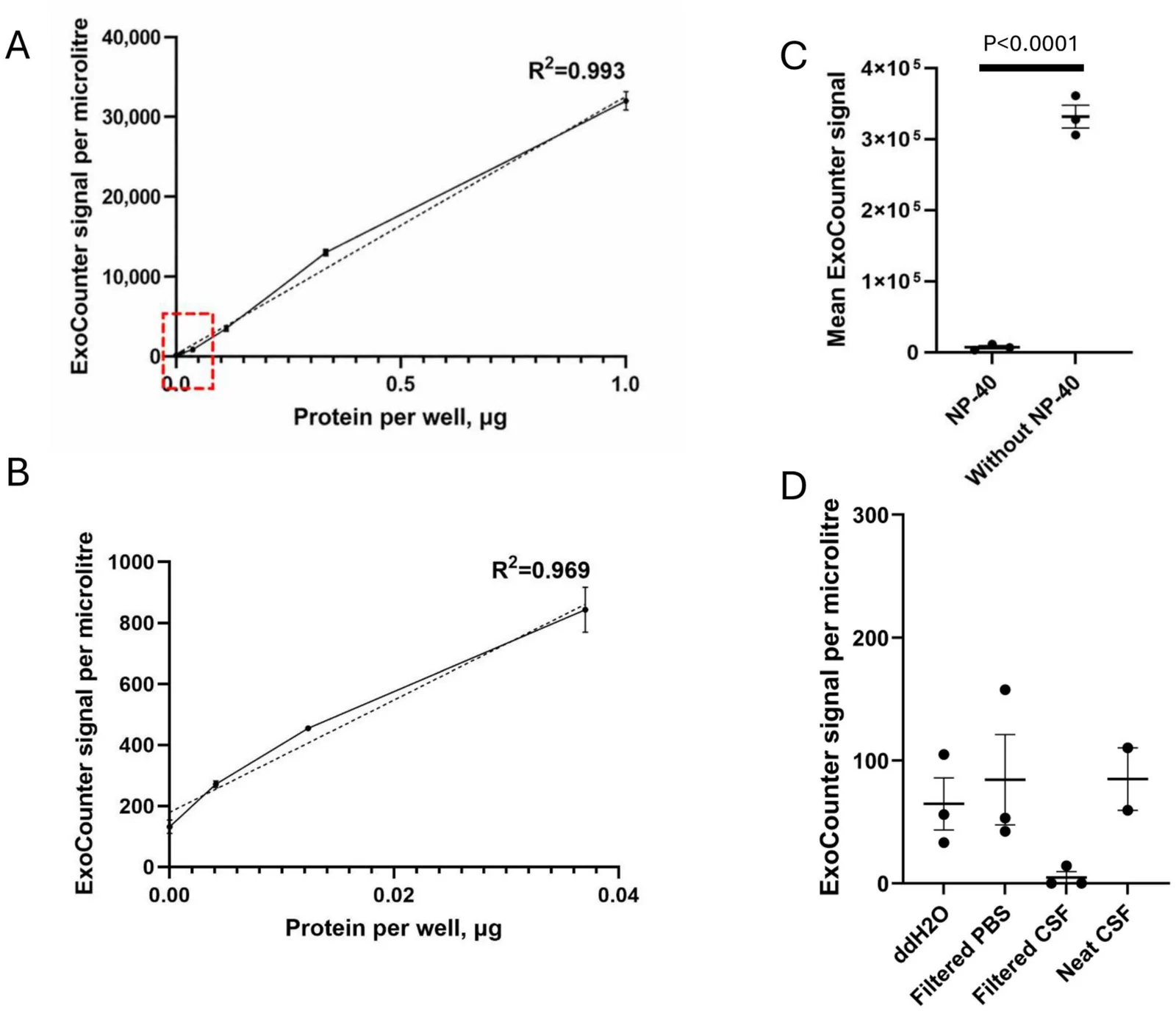
Quantification of psEVs in non-pregnant CSF following spike-in of isolated psEVs. (**A**) Graph showing the mean ExoCounter values for different amounts of spiked-in psEVs in non-pregnant CSF. Error bars represent standard deviation. (**B**) Enhancement of the same graph as (**A**) but showing only the lower values as indicated by the red box. (**C**) The ExoCounter signal from a sample pre-treated with or without NP-40. Bars represent mean and SEM. (**D**) ExoCounter signal per microlitre for double-distilled water, filtered PBS, ultrafiltered CSF (10kDa molecular weight cut-off membrane) and neat CSF.

To determine whether ExoCounter assay linearity is maintained with CSF, linearity testing was performed by spiking isolated psEVs into non-pregnant CSF. Measured sEV counts were compared to the known protein concentrations of isolated psEV, as determined by bicinchoninic acid (BCA) assay. Linearity of psEVs spiked into CSF was high (R^2^ = 0.994) and remained high at very low levels of spiked protein (Figure 2A,B). psEVs could be clearly detected at 0.00412 μg of spiked protein per well (determined by BCA assay), equivalent to <0.00009 μg of sEV protein per microlitre of sample. These results suggest that the ExoCounter assay is suitable for use in CSF samples and can detect PLAP+ CD63+ sEVs present at very low levels in CSF.

**Figure 3:**
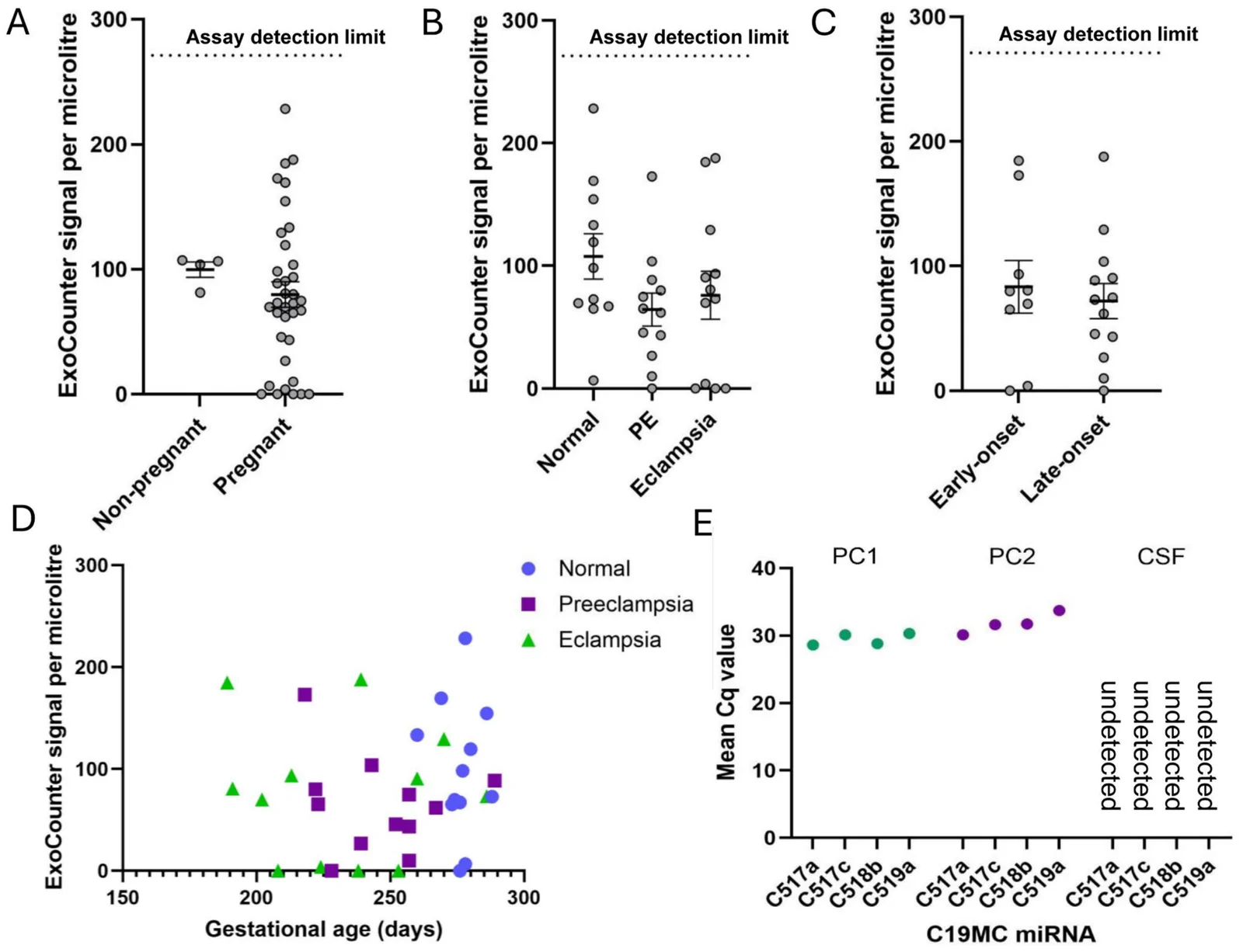
Quantifying psEVs in CSF samples of preeclamptic, eclamptic, normotensive pregnancies and non-pregnant women. (**A**) ExoCounter signals from non-pregnant and pregnant CSF samples. Bars represent mean and SEM. The dotted line represents the limit of detection from our linearity testing in spiked CSF samples. (**B**) ExoCounter signals from patients in different clinical groups. (**C**) ExoCounter signals from patients with early- and late-onset preeclampsia/eclampsia. (**D**) Scatter plot showing ExoCounter signal against gestational age, grouped by clinical presentation. (**E**) Mean Cq values from RT-qPCR for four miRNAs of the C19 cluster. PC1 and PC2 are positive controls (isolated psEVs). CSF samples with sufficient volume remaining were tested *(n* = 9), with miRNAs being undetectable in all CSF samples. The nine CSF samples are thus represented by one data point per miRNA.

Non-pregnant CSF samples (*n* = 4) were run on the ExoCounter to measure the baseline signal when psEVs are not present. Non-pregnant CSF samples had a mean signal of 98.7 ± 12.3 (mean ± SD) counts per microlitre (Figure 3A). Signals obtained from a neat non-pregnant CSF sample, double-distilled water and filtered PBS run on the same day were similar (84.9, 64.7 and 84.4 counts per microlitre respectively), but ultrafiltration of the same non-pregnant CSF sample through a 10 kDa MWCO membrane resulted in a signal decrease to 4.8 counts per microlitre (Figure 2D). Isolated psEVs were used to check dependence of the ExoCounter assay on intact vesicular structure. psEVs were exposed to NP-40, a surfactant which disrupts lipid membranes. Comparison revealed a significant loss of signal in the NP-40-treated sample (Figure 2C, *p* < 0.0001).

### 3.2. Measuring psEVs in the CSF of Women with Normotensive Pregnancies, Preeclampsia and Eclampsia

Between March and November 2021, CSF samples from 33 pregnant women were included in our study. The samples were divided into three clinical groups: normotensive pregnancies (*n* = 11), preeclamptic pregnancies (*n* = 12) and eclamptic pregnancies (*n* = 10). Clinical and demographic characteristics of participants are shown in Table 1. Compared with normotensive pregnant women, women with preeclampsia or eclampsia were more likely to be primiparous, deliver at an earlier gestational age, have an emergency caesarean section, severe hypertension, pulmonary oedema, HELLP (hemolysis, elevated liver enzymes, and low platelet count) syndrome and be admitted to the obstetric critical care unit (OCCU).

**Figure 4:**
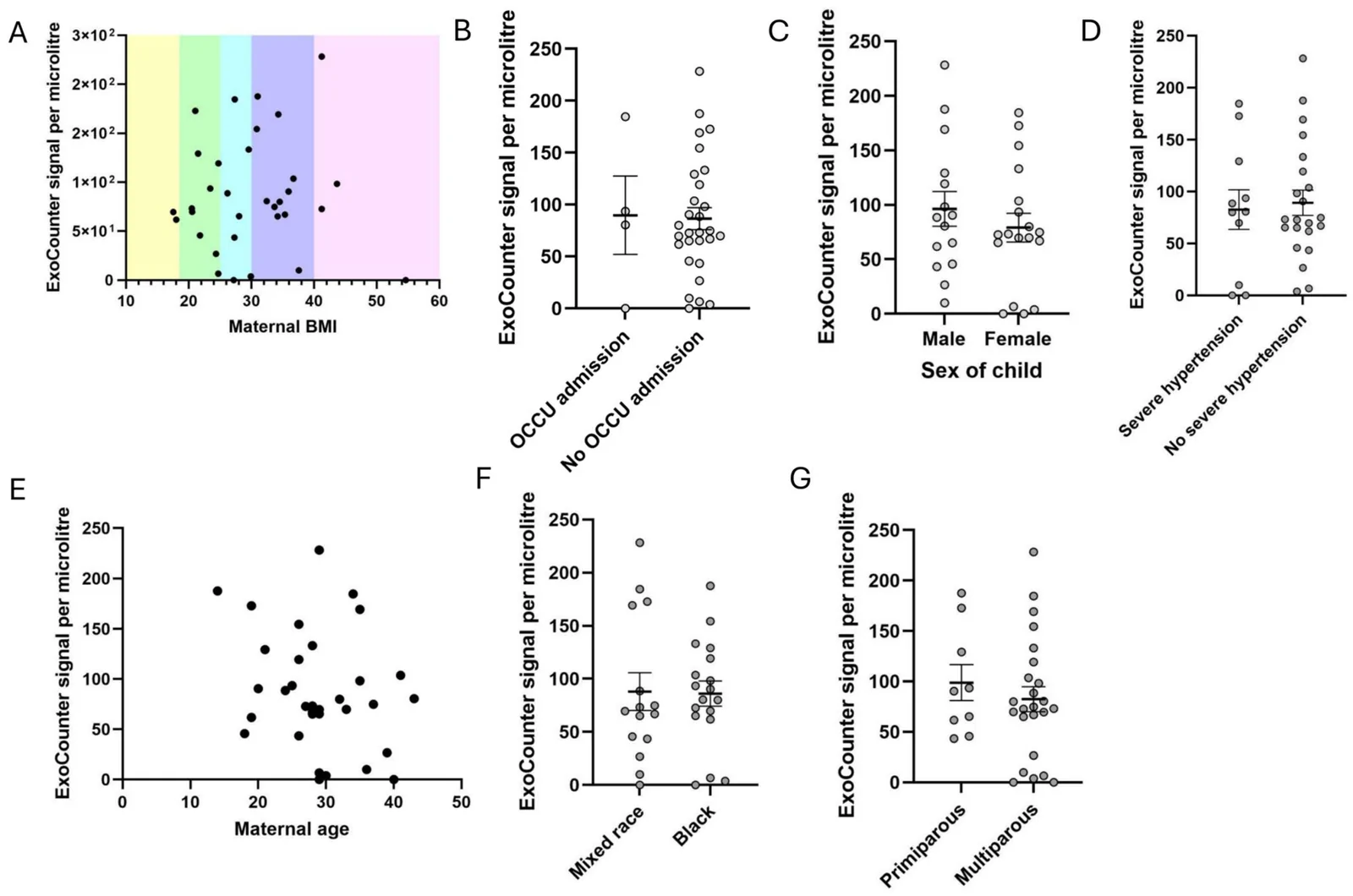
ExoCounter signals by participant groupings, considering clinical characteristics. (**A**) ExoCounter signal by patient BMI. Colours correspond to BMI categories: yellow for underweight, green for healthy weight, blue for overweight, purple for obese and pink for severely obese. No correlations were observed with maternal BMI. (**B**) ExoCounter signal presented by OCCU admission; (**C**) sex of child; (**D**) occurrence of severe hypertension; (**E**) maternal age; (**F**) maternal ethnicity; and (**G**) parity. For each graph, dots represent individual participants, while bars represent mean ± SEM.

The mean ExoCounter signal for CSF samples from pregnant participants was 79.7 ± 60.8 counts per microlitre. No difference was observed between the pregnant and non-pregnant groups (Figure 3A). There were no differences in ExoCounter signal between clinical groups, regardless of early-onset versus late-onset disease (Figure 3B,C). There was no association between the ExoCounter signal in CSF and gestational age at time of sampling (Figure 3D). There were no associations between ExoCounter signal and maternal BMI, maternal age, admission to OCCU, sex of child, severe hypertension, maternal ethnicity, emergency C-section, or primiparity (Figure 4A–G). Information on magnesium sulfate treatment was missing for the majority of participants, but four participants were confirmed not to have been treated with it and showed similarly low ExoCounter counts (51.9 ± 61.8).

RNA-based techniques were used to verify the lack of psEV signal in nine CSF samples with sufficient volume remaining (four samples from normotensive pregnancies, one from preeclamptic pregnancy and four from eclamptic pregnancies). These were interrogated for presence of the placenta-specific chromosome 19 (C19) miRNA cluster using qPCR. Two aliquots of psEVs were used as positive controls (PC1 and PC2). Nanodrop and Qubit measurements demonstrated the presence of RNA and miRNA in CSF following RNA isolation and cDNA following cDNA synthesis (Table 2). C19 miRNAs were detected in both positive controls but in none of the CSF samples (Figure 3E).

## 4. Discussion

psEVs were not detectable in the CSF of women with normotensive, preeclamptic nor eclamptic pregnancies using a highly sensitive psEV quantification assay and comparison to CSF from non-pregnant donors. This was despite assay linearity testing showing discernible signal even at very low levels of psEVs. The placenta-associated C19 miRNA cluster was also undetected in pregnant CSF samples.

Low-level ExoCounter signal was present in both pregnant and non-pregnant samples, below the limit of detection, as determined by linearity testing. The signal was comparable to the signal obtained from filtered PBS or double-distilled water and was consistent across temporally distant runs, suggesting that this background signal may be due to non-biological contaminants, such as dust or other undetermined small particulates. Ultrafiltration of a non-pregnant CSF sample through a 10 kDa molecular weight cut-off membrane reduced this low-level signal from 84.9 to 4.8 counts per microlitre, supporting the idea that low-level signal may be due to small particulates/contaminants present in samples, most likely of non-biological origin, given that a similar signal is observed in double-distilled water and filtered PBS.

This study contributes new information on the biodistribution of psEVs in human pregnancy and is the first to search for psEVs in human neurological tissue. It is well-established that psEVs circulate in maternal plasma but biodistribution of these EVs amongst maternal organs has not been determined. In vitro evidence strongly supports the capacity of psEVs to influence recipient cells, but it is difficult to translate in vitro effects into an in vivo context without understanding psEV localisation.

Several mouse studies attempt to address this knowledge gap, typically by injection of a large bolus of traceable human psEVs into the tail vein [34,35]. This methodology fails to recapitulate the continuous low-level release of psEVs in vivo and may result in unrepresentative accumulation of psEVs into major clearance organs. Furthermore, the mismatch between human psEVs and the mouse host is likely to impact localisation, both because of immune responses and the importance of interactions between psEV surface proteins and recipient cells in determining psEV tropism [36]. Thus, it is difficult to extrapolate findings from mouse biodistribution studies into a physiological human context [37].

This study found no evidence of psEVs in the CSF, but it is important to note limitations. Firstly, the sample sizes are small, particularly for the eclampsia group. Furthermore, while there was some range in gestational age at the time of sampling (range: 27–41 + 2 gestational weeks), almost all participants were in the third trimester, so no conclusions can be drawn about the first and second trimesters. Samples were taken from a single timepoint, so transient presence of psEVs in CSF may be missed, for example, immediately preceding or during seizure. Although Jiang et al. identified CD63 and PLAP as the most suitable marker combination for psEV identification with the ExoCounter assay, some psEVs will not co-express these markers and will thus be missed by the assay [13].

It is possible that psEVs may be present in CSF at levels too low for detection by our assay. Indeed, it is well-established that CSF generally has a very low EV content compared to other biofluids [38,39]. Due to limited sample volumes, it was not possible to concentrate CSF sEVs before the assay. Another study with access to larger CSF volumes may wish to concentrate sEVs from pregnant and non-pregnant CSF before quantification. Furthermore, it is possible that psEVs were present in the initial sample but were degraded in sample processing or the single freeze–thaw cycle, although evidence suggests that EVs carried in CSF are relatively robust to freeze–thaw cycles and degradation [40].

This study does not rule out the possibility that psEVs reach the CSF but are rapidly taken up by surrounding tissues, thus preventing detection in CSF. One study found that the half-life of EVs injected intrathecally into CSF was shorter than EVs in plasma [41]. It is not clear to what extent this is due to uptake by brain tissues, diffusion into plasma, or EV instability. Cell-based studies may be used to determine the capacity of neurological cell types to uptake psEVs.

This study does not necessarily contradict previous studies showing that psEVs disrupt BBB monolayer models in vitro, as psEVs may directly interact with BBB tissue, without crossing into CSF or brain parenchyma. Endothelial cells form the structural core of the BBB [42], and there is extensive evidence of preeclamptic psEVs inducing endothelial dysfunction and inhibiting endothelial cell proliferation [15,16,17]. Blocking uptake of preeclamptic psEVs protects human vascular endothelium from structural damage [18].

Recent publications demonstrate that preeclamptic sEVs increase vascular permeability and disrupt integrity of BBB monolayer models through disruption of endothelial cell–cell junctions [29,43]. Notably, most previous studies on the effects of psEV exposure on BBB models tend to use trans-endothelial electronic resistance, small solutes, or expression of tight-junction proteins as markers of BBB permeability, and do not examine permeability of the membrane to sEVs [28,29,44]. These studies provide proof-of-concept evidence that preeclamptic psEVs increase BBB permeability, but do not address barrier permeability to psEVs specifically.

Preeclamptic psEVs may exert indirect effects on the BBB by promoting a systemic pro-inflammatory state, which in turn impairs BBB function [45,46]. Preeclamptic psEVs promote macrophage polarisation towards an inflammatory state [17,19,20], modulate lymphocyte and monocyte responses [17,21,46], and may directly induce inflammation in endothelial cells [17,22]. Several studies show that BBB permeability is increased in response to systemic inflammation due to disrupted tight junctions [45].

Overall, this study did not find evidence to support the presence of psEVs in the CSF of pregnant women, regardless of preeclampsia or eclampsia occurrence. This aligns with the findings of some mouse models, which did not see psEV uptake into mouse brains [34,35]. Although there are limitations with both mouse biodistribution studies and our own human tissue studies, both lines of evidence suggest that psEVs may not cross from peripheral circulation into CSF. The contribution of psEVs to preeclamptic neuropathology may be mediated indirectly via induction of systemic inflammation and endothelial dysfunction, or by direct interaction of psEVs with cells of the BBB or blood–CSF barrier, without psEVs crossing these barriers.

## 5. Conclusions

Little is known about the biodistribution of psEVs in pregnant women. This study tested the hypothesis that psEVs may be present in CSF and contribute to neurological symptoms of preeclampsia. Using a highly sensitive sEV quantification assay and RNA-based sEV detection techniques, psEVs were not detected in the CSF of pregnant women with normotensive, preeclamptic or eclamptic pregnancy. This is the first study to search for psEVs in human central nervous tissue and represents a step towards understanding psEV biodistribution in pregnancy.

## Figures and Tables

**Table 1:** Clinical characteristics of participants.

	Total, *n* = 33	Normotensive, *n* = 11	Preeclamptic, *n* = 12	Eclamptic, *n* = 10	*p*-Value
Mean gestational age at birth (days) ± SD	252 ± 29.0	276 ± 7.7	246 ± 21.2	231 ± 33.5	<0.001
Mean pre-pregnancy maternal BMI ± SD	29.9 ± 8.3	32.5 ± 8.0	27.8 ± 6.5	29.7 ± 10.3	0.410
Mean maternal age ± SD	29.0 ± 7.0	29.1 ± 3.1	29.1 ± 8.1	28.8 ± 9.1	0.997
Primiparous	27%	0%	42%	40%	0.040
Mean gravidity ± SD	2.85 ± 1.8	3.55 ± 1.1	2.25 ± 1.2	2.80 ± 2.6	0.212
Female child	55%	55%	50%	60%	0.896
HIV positive	9%	9%	8%	10%	1.000
Early-onset (delivery < 34 weeks)	24%	N/A	25%	50%	0.210
Intrauterine death	2	0	0	2	0.085
Severe hypertension	33%	0%	50%	60%	0.007
Emergency C-section	52%	9%	58%	90%	<0.001
Pulmonary oedema	9%	0%	0%	30%	0.022
HELLP syndrome	9%	0%	0%	30%	0.022
Admitted to obstetric critical care unit	14%	0%	0%	40%	0.005

BMI: body mass index; SD: standard deviation; HELLP syndrome: haemolysis, elevated liver enzymes and low platelet syndrome.

**Table 2:** Nanodrop and Qubit results for isolated RNA.

Sample	Nanodrop	Qubit		
	RNA Conc., ng/μL	RNA Conc., ng/μL	miRNA Conc., ng/μL	cDNA Conc., ng/μL
CSF1	34.9	8.7	183	0.53
CSF2	23.0	6.7	246	0.53
CSF3	24.4	6.9	156	0.65
CSF4	42.2	7.1	210	0.57
CSF5	31.1	7.2	120	0.58
CSF6	32.3	7.7	156	0.60
CSF7	22.3	5.8	149	0.59
CSF8	18.2	5.5	160	0.53
CSF9	20.4	5.7	156	0.57
Positive Control 1	41.6	12.6	988	0.55
Positive Control 2	65.5	14.9	1252	0.55

## Data Availability

The raw data supporting the conclusions of this article will be made available by the authors upon request.

## Supplementary Materials

The following supporting information can be downloaded at: https://www.mdpi.com/article/10.3390/biom16081211/s1, All original images.

## Abbreviations

The following abbreviations are used in this manuscript:

(p)sEV	(placental) small extracellular vesicle
C19	chromosome 19
PLAP	placental alkaline phosphatase
PBS	phosphate-buffered saline
TEM	transmission electron microscopy
NTA	nanoparticle tracking analysis
BCA	bicinchoninic acid
NP-40	nonidet P-40
OCCU	obstetric critical care unit
HELLP	haemolysis, elevated liver enzymes and low platelet count
CSF	cerebrospinal fluid
BBB	blood-brain barrier
