# Supplementary material for: Placental Small Extracellular Vesicles Undetected in Cerebrospinal Fluid of Preeclamptic and Eclamptic Women
Source: Biomolecules. 2026 Aug 19;16(8):1211. doi: 10.3390/biom16081211 (PMC13511577; doi:10.3390/biom16081211)

Uncropped blots and Ponceau red

1A

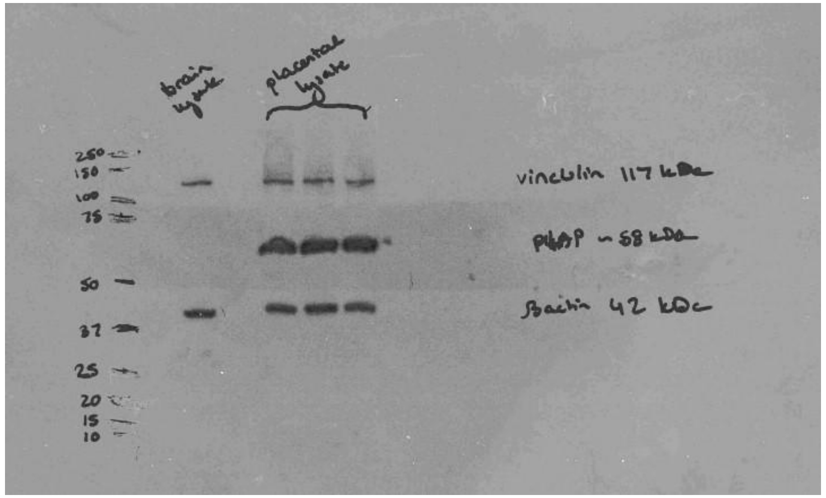

1B

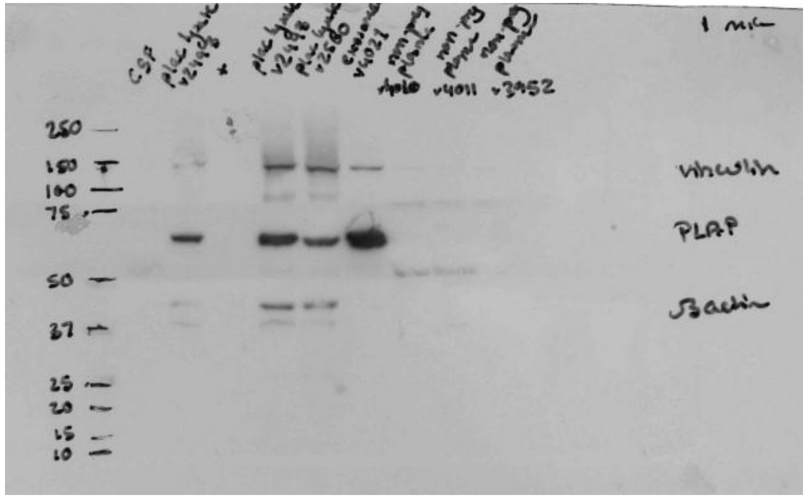

1A

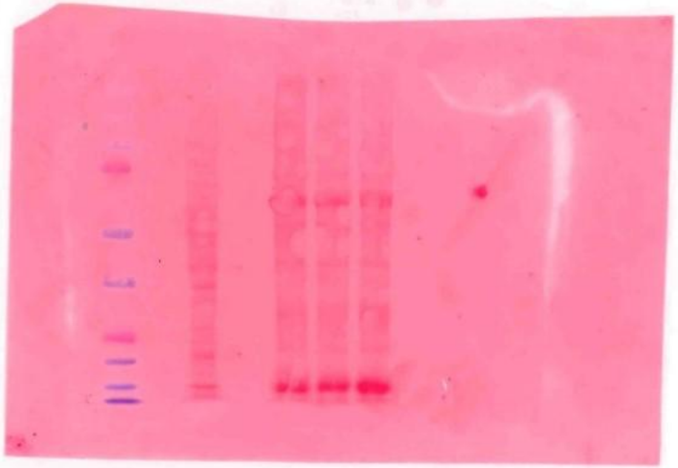

1B

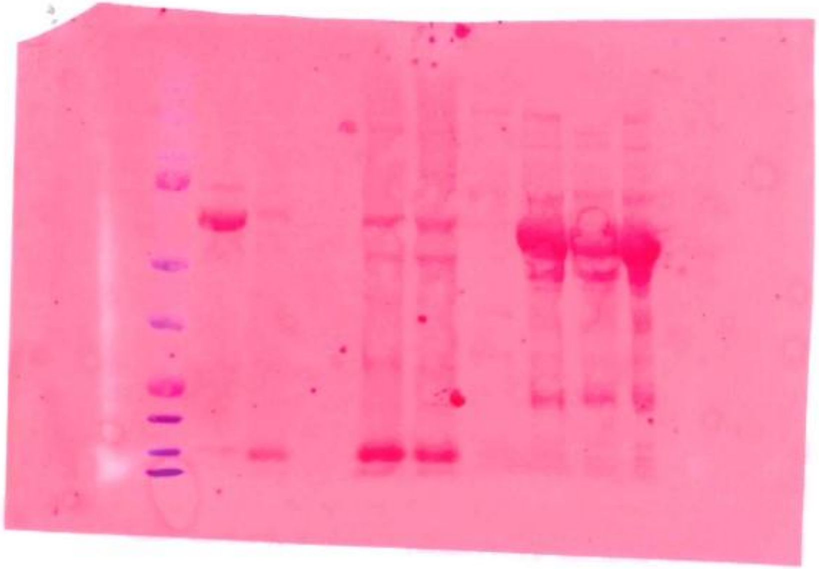

Uncropped blots

CD63

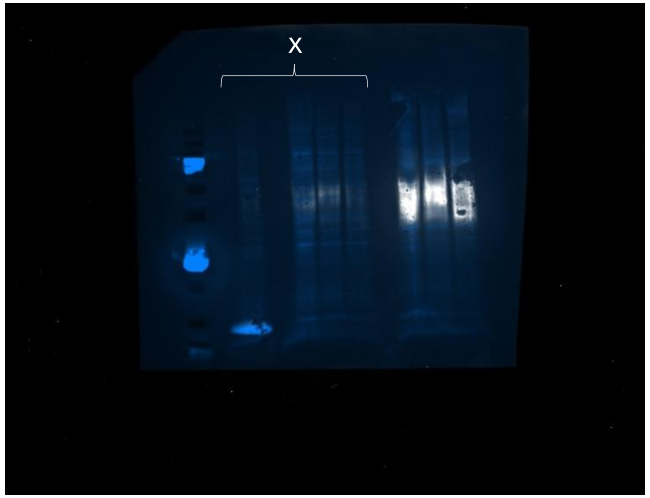

Syntenin and cytochrome c

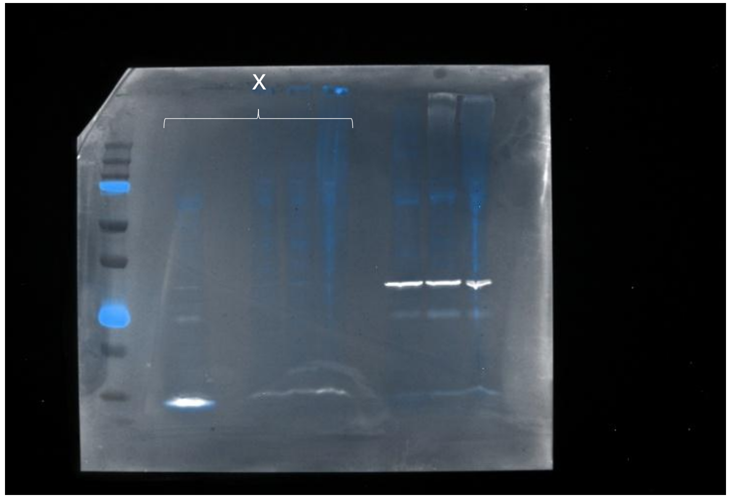

Alix

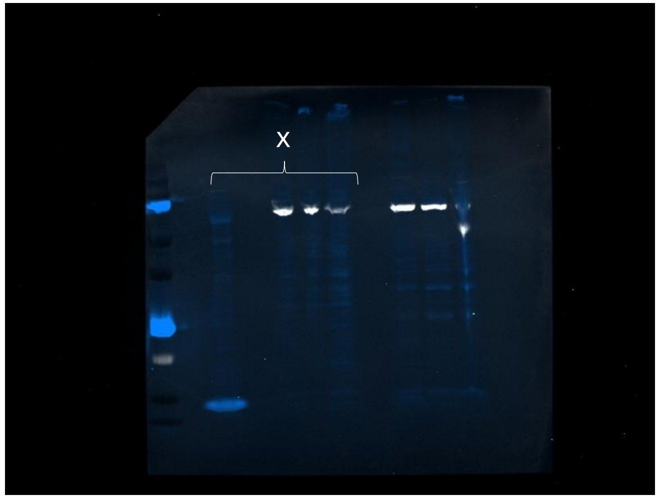

x=these lanes were part of another experiment and not included in our manuscript

As above, labelled

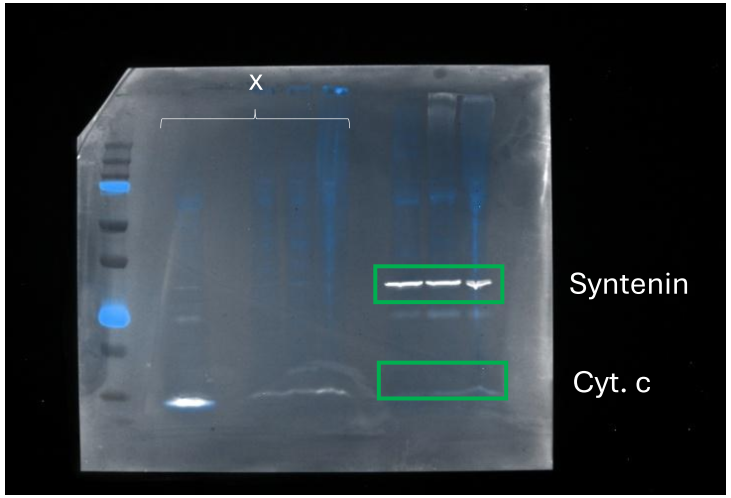

TEM images

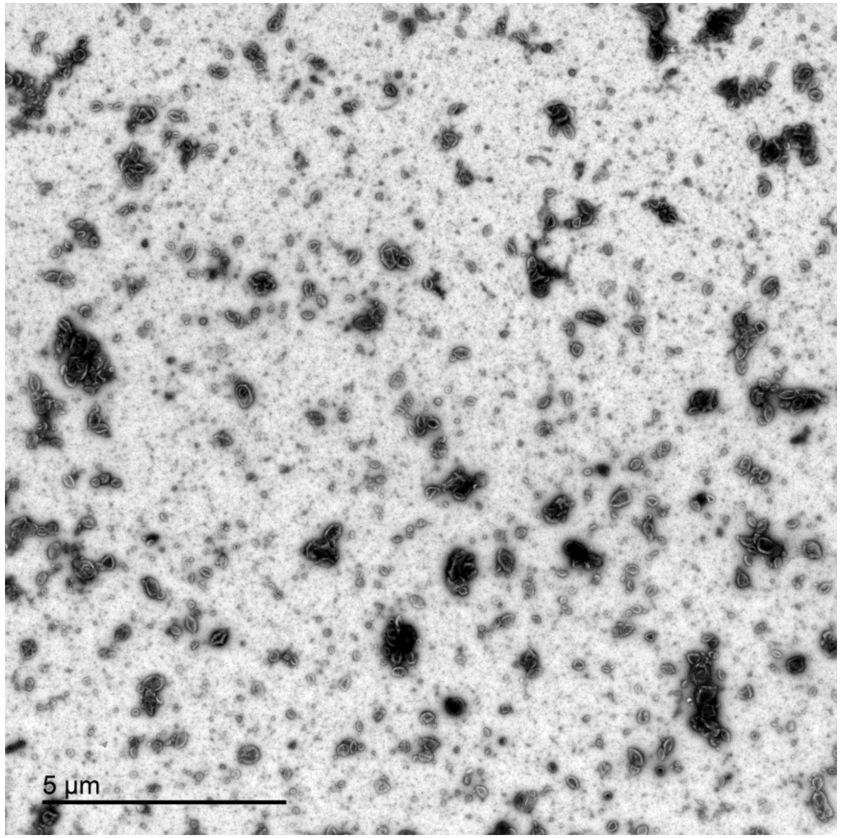

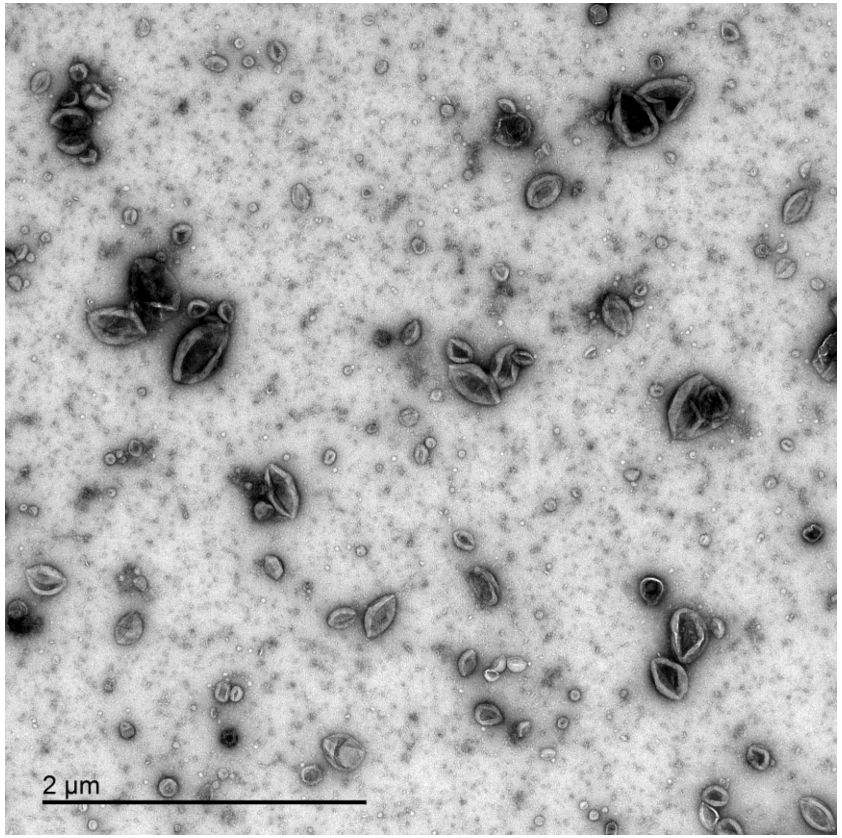

Supplement: Supplementary file 1 [file biomolecules-16-01211-s001.zip › biomolecules-4439909-supplementary.pdf]
